# High-resolution MRI of flexor tendon pulleys using a 16-channel hand coil: disease detection and differentiation of psoriatic and rheumatoid arthritis

**DOI:** 10.1186/s13075-020-2135-0

**Published:** 2020-03-02

**Authors:** Daniel B. Abrar, Christoph Schleich, Sven Nebelung, Miriam Frenken, Karl Ludger Radke, Stefan Vordenbäumen, Ralph Brinks, Matthias Schneider, Benedikt Ostendorf, Dennis McGonagle, Philipp Sewerin

**Affiliations:** 1 Institute for Diagnostic and Interventional Radiology, UKD, Heinrich Heine University Düsseldorf, Moorenstrasse 5, 40225 Düsseldorf, Germany; 2 Policlinic and Hiller Research Unit for Rheumatology, UKD, Heinrich Heine University Düsseldorf, Moorenstrasse 5, 40225 Düsseldorf, Germany; 3 NIHR Leeds Musculoskeletal Biomedical Research Unit, Leeds Teaching Hospitals Trust and The University of Leeds, Leeds, UK

**Keywords:** Psoriatic arthritis, Pulley, PsAMRIS, Enthesitis, Rheumatoid arthritis, Synovio-entheseal-complex

## Abstract

**Background:**

To evaluate the value of 3 Tesla (T) magnetic resonance imaging (MRI) changes of flexor tendon pulleys for the differentiation of psoriatic (PsA) and rheumatoid arthritis (RA), using a novel 16-channel high-resolution hand coil.

**Methods:**

Seventeen patients with active PsA, 20 patients with active RA, and 16 healthy controls (HC) underwent high-resolution 3 T MRI using the dedicated 16-channel hand coil. Images were analyzed by three independent readers for the degree of inflammatory changes, thickness of flexor tendon pulleys, and comparison to the outcome measures for RA clinical trials (OMERACT) PsA MRI score (PsAMRIS) and to its sub-scores. For correlation analyses, Spearman rho correlation was calculated.

**Results:**

Flexor tendon pulleys were thicker in PsA than in RA patients (mean difference 0.16 mm, *p* < 0.001) and HC (mean difference 0.2 mm, *p* < 0.001) and showed a higher degree of associated inflammatory changes (mean difference from RA 4.7, *p* = 0.048; mean difference from HC 14.65, *p* < 0.001). Additionally, there was a strong correlation of accessory pulley inflammation and PsAMRIS and its acute-inflammatory sub-scores, flexor tenosynovitis, synovitis, and periarticular inflammation (for the second digit synovitis *ρ* = 0.72, flexor tenosynovitis *ρ* = 0.7, overall PsAMRIS *ρ* = 0.72, *p* < 0.01). Similar robust correlations were evident in digits 3–5. Weaker correlations were evident in RA (synovitis *ρ* = 0.49, flexor tenosynovitis *ρ* = 0.49, periarticular inflammation *ρ* = 0.4).

**Conclusion:**

The assessment of MRI changes of flexor tendon pulleys is potentially beneficial for disease detection in PsA, as well as for its distinction from RA and HC.

**Trial registration:**

2014123117, December 2014.

## Background

Psoriatic arthritis (PsA) is a very common chronic inflammatory disease that affects joints and ultimately leads to joint destruction and functional disabilities [1, 2]. Regarding its clinical presentation, PsA shares many similarities with rheumatoid arthritis (RA), which potentially complicates the distinction between both entities, especially in cases of symmetric and seronegative RA [3–5]. However, PsA and RA differ in their pathophysiology: RA is considered to be a synovial disease that exhibits secondary spread to the adjacent bone; PsA on the other hand characteristically affects entheses, such as tendon and ligament insertion sites. These can be classified as fibrous and fibrocartilaginous and belong to the so-called synovio-entheseal complex [6–10]. Additionally, tendons build so-called functional entheses with associated ligamentous structures, such as flexor tendon pulleys [11]. These pulleys have a fibrocartilaginous component and discontinuously wrap around the flexor tendons preventing their bowstringing during flexion [12]. Recent studies have shown that inflammation and thickening of flexor tendon pulleys are potentially due to mechanical stress (“deep Koebner response”) and may lead to the initial development of flexor tenosynovitis and dactylitis, that are major features of PsA [13–15].

As in RA, remission is the ultimate goal of disease-modifying therapy in PsA. Hence, early accurate diagnosis and treatment are pivotal for a favorable clinical outcome [16]. Additionally, distinct pharmacological options and treat-to-target strategies exist for both diseases which underlines the importance of an unequivocal distinction [3, 17, 18]. Even though not yet included in the Classification Criteria for PsA (CASPAR), magnetic resonance imaging (MRI) is becoming increasingly important and widely used for diagnosis and the monitoring of therapy for PsA [19–21]. The Outcome Measures for Rheumatoid Arthritis Clinical Trials (OMERACT) working group has developed a semi-quantitative PsA MRI (sum-)score (PsAMRIS) that is highly sensitive for disease-related joint changes and is widely used for therapy monitoring [22–24]. However, until most recently, enthesitis was not included in any MRI score, despite being a hallmark of PsA [25, 26]. Indeed, enthesitis is difficult to accurately evaluate in small joints using conventional MRI or with high-resolution MRI surface coils that can only visualize a small area. We addressed this by using a dedicated 16-channel high-resolution hand coil to permit a global evaluation of digits in PsA and RA. Herein, we describe our findings indicating that this technique has considerable potential to differentiate between RA and PsA hand involvement.

## Methods

### Patients

Seventeen patients (mean age 53.7 ± 11.6; minimum/maximum 26/72 years, male/female 9/8) fulfilling the CASPAR criteria with a mean disease duration of 2.6 ± 3.3 years and peripheral joint involvement and dactylitis were prospectively recruited for the “Analysis of the DActylic Melange” (ADAM) research initiative. All patients had failed methotrexate (MTX) monotherapy and were escalated to Etanercept (Enbrel® 50 mg s.c. fortnightly) after a baseline MRI scan.

Additionally, 20 therapy naïve patients (mean age 46 ± 15.7, minimum/maximum 19/67 years, male/female 9/11), fulfilling the ACR/EULAR 2010 criteria for RA with a mean disease duration < 6 months (mean duration 11 ± 7 weeks) from the “Cartilage in early RA” (CAR-ERA) study, were included. Patients were allowed a daily dose of oral prednisone at < 10 mg. After a baseline scan, patients received either MTX monotherapy or a combination of MTX and adalimumab. Patients were blinded for their therapy regime. PsA and RA patient characteristics are visualized in Table 1.

**Table 1:** Characteristics of the study population. For patients with psoriatic arthritis (PsA) and rheumatoid arthritis (RA), as well as healthy controls the population size, the mean age in years ± standard deviation and range, the mean disease duration (except HC) in years (PsA) and weeks (RA) ± standard deviation and range and the sex are presented

	PsA patients	RA patients	HC
Population size	17	20	16
Age [years]	53.7 ± 11.6 (26–72)	46 ± 15.7 (19–67)	39 ± 16.1 (range 17–78)
Disease duration	2.6 ± 3.3 (1–8 years)	11 ± 7 (2–24 weeks)	–
Sex [male/female]	9 males/8 females	11 males/9 females	9 males/7 females

Furthermore, 16 patients (mean age 39 ± 16.1, minimum/maximum 17/78 years, male/female 9/7) with no history of arthritis were retrospectively recruited as healthy controls (HC). MRI studies were performed due to clinical reasons (e.g., suspected carpal ganglion) in our daily routine. At the time of retrospective recruitment, all subjects of HC were over 18 years of age. The study was approved by the local ethics committee (MO-LKP-719, 4962R). Written and informed consent was obtained from all patients before initiation of the study.

### MR imaging

**Fig. 1:**
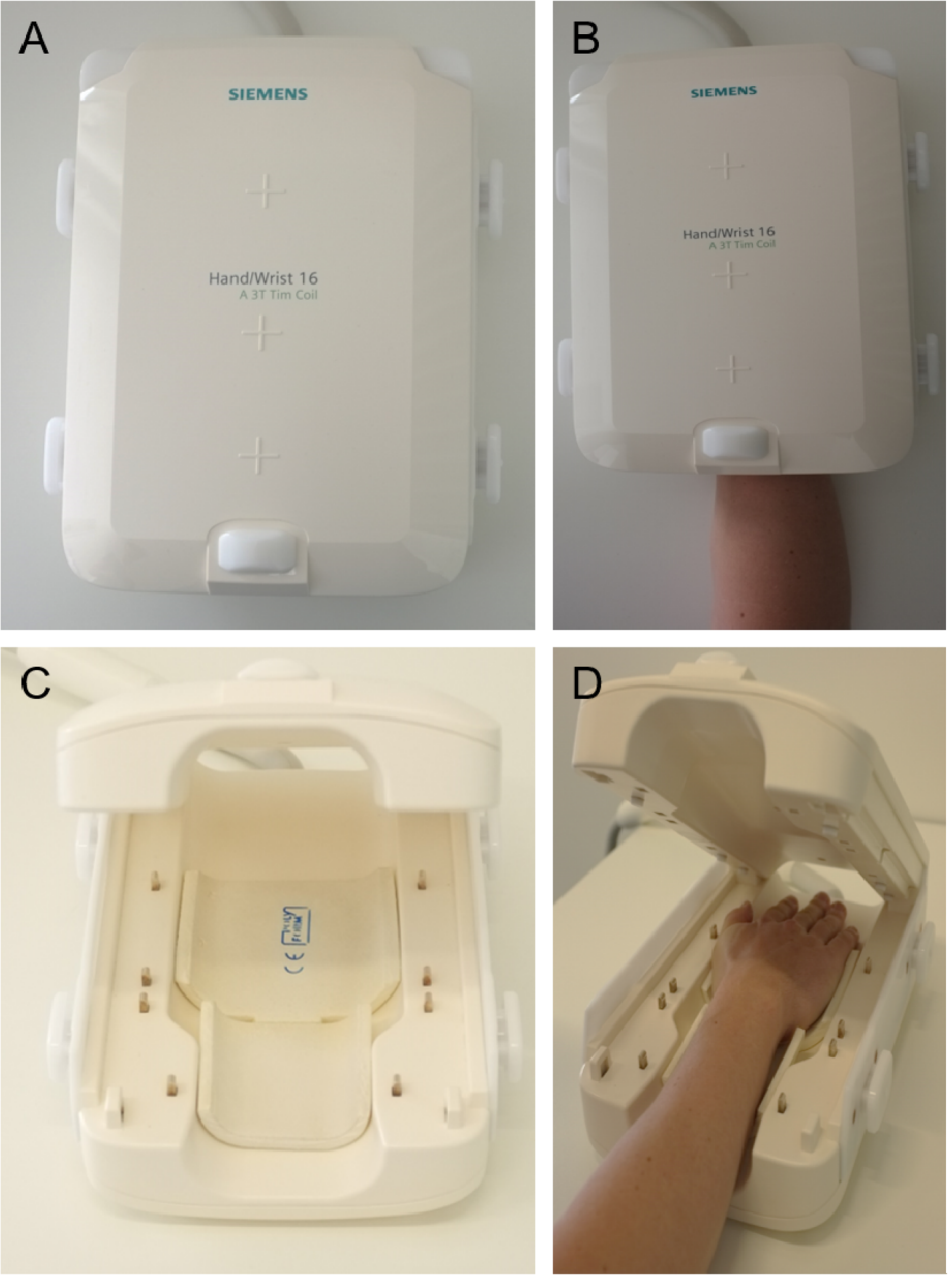
16-channcel hand coil

For MR imaging, a 3 T MRI scanner (Magnetom Skyra, Siemens Healthineers, Erlangen, Germany) and a dedicated 16-channel hand coil (Fig. 1; 3 T Tim Coil, Siemens Healthineers, Erlangen, Germany) were used, allowing for a high-resolution imaging over a wide area. PsA patients received a baseline (T0) and a follow-up (T1) scan with an approximately 6.2 ± 0.85 months (minimum/maximum: 5/8 months) interval in between. A baseline (T0) and two follow-up (T1 and T2) scans were performed in the RA population with approximately 2.8 ± 0.1 months (minimum/maximum 2.6/3 months) between T0 and T1 and 5.6 ± 0.1 months (minimum/maximum 5.4/5.8 months) between T0 and T2. For HC only a single scan was performed. In patients, all baseline scans were used for further image analysis.

The imaging protocol followed the recommendations of the OMERACT working group for PsA and RA [21, 27]. In PsA patients this included pre- and post-contrast (DOTA^−^, Dotarem, Guerbet, Villepinte, France; intravenous-injection of 0.4 ml/kg bodyweight) T1-weighted turbo spin echo (TSE) and non-contrast-enhanced fat-saturated T2-weighted TSE or short tau inversion recovery (STIR) sequences in two different orthogonal planes. The field of view covered MCP, PIP and DIP of digits 2–5. In RA patients the protocol included the following sequences: pre- and post-contrast (DOTA^−^, Dotarem, Guerbet, Villepinte, France intravenous-injection of 0.4 ml/kg bodyweight) coronal T1-weighted TSE and transversal fat-saturated T1-weighted spin echo (SE) after contrast agent application as well as a coronal STIR. The field of view covered MCP 2–5, carpometacarpal, carpal, radiocarpal, and distal radioulnar joints.

In control patients, our in-house standard protocol was used, which included the same sequences as detailed for the RA patients above. In addition, a transversal fat-saturated proton-density-weighted sequence was acquired. The field of view differed according to the clinical region of interest. All participants were scanned in the prone position with their clinically dominant hand extended overhead and the palm facing down (“superman position”).

The sequence parameters were as follows:

PsA: coronal T1 TSE (TR/TE in ms, PsA: 862/27, RA: 862/27; flip angle in °, PsA: 150, RA: 150; slice thickness in mm, PsA: 2.5, RA: 2.5; field of view in mm, PsA: 140, RA: 130; pixel size: PsA: 0.3 × 0.3 mm, RA: 0.3 × 0.3; acquisition matrix: 512 × 512), coronal STIR (TR/TE in ms, PsA: 5560/31, RA: 5560/31; flip angle in °, PsA: 120, RA: 120; slice thickness in mm, PsA: 2.5, RA: 2.5; field of view in mm, PsA: 140, RA: 130; pixel size: PsA: 0.3 × 0.3 mm, RA: 0.3 × 0.3 mm; acquisition matrix: 448 × 314), sagittal PD TSE fat-saturated (only PsA: TR/TE in ms 3150/47, flip angle 150°, slice thickness 2.5 mm, field of view 150 mm; pixel size: 0.3 × 0.3 mm; acquisition matrix: 448 × 182), transversal T2 TSE fat-saturated (only PsA: TR/TE in ms: 5693.8/89, flip angle 180°, slice thickness 3.0 mm, field of view: 160 mm; pixel size: 0.3 × 0.3 mm; acquisition matrix: 512 × 358), transversal T1 SE fat-saturated after iv contrast (TR/TE in ms, PsA: 807/16, RA: 702/16; flip angle in °, PsA: 90, RA: 90; slice thickness in mm, PsA: 3.0, RA 2.5; field of view in mm, PsA:130, RA: 120; pixel size: PsA: 0.3 × 0.3 mm, RA: 0.3 × 0.3 mm; acquisition matrix: 384 × 288) and coronal T1 TSE after iv contrast (TR/TE in ms, PsA: 862/27, RA: 862/27; flip angle in °, PsA: 150, RA; 150; slice thickness in mm, PsA: 2.5, RA: 2.5; field of view in mm, PsA; 140, RA: 140; pixel size: PsA: 0.3 × 0.3 mm, RA: 0.3 × 0.3 mm; acquisition matrix: 512 × 512).

### Image analysis

**Fig. 2:**
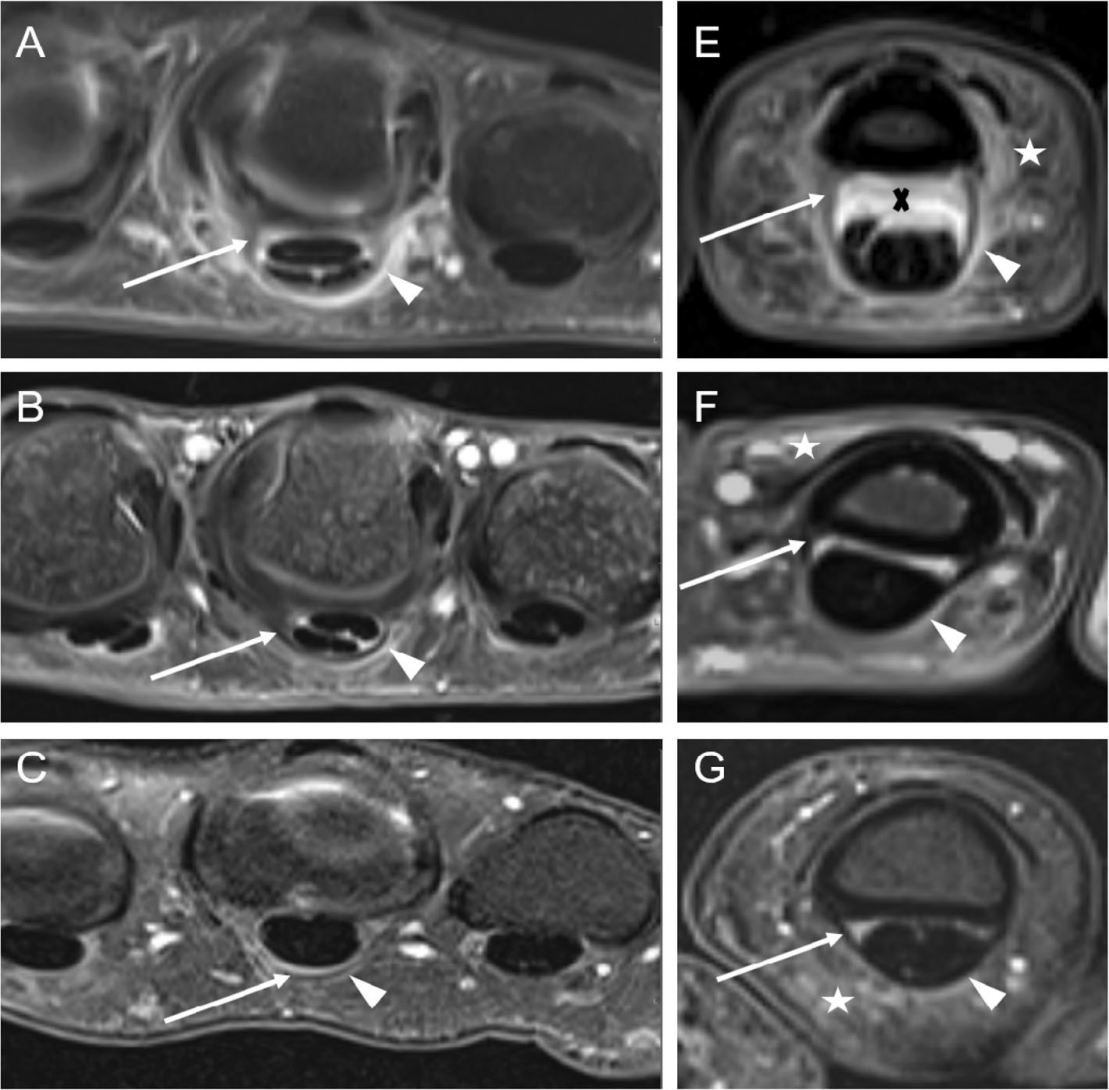
High-resolution MRI of the Hand - Figure 2

**Fig. 3:**
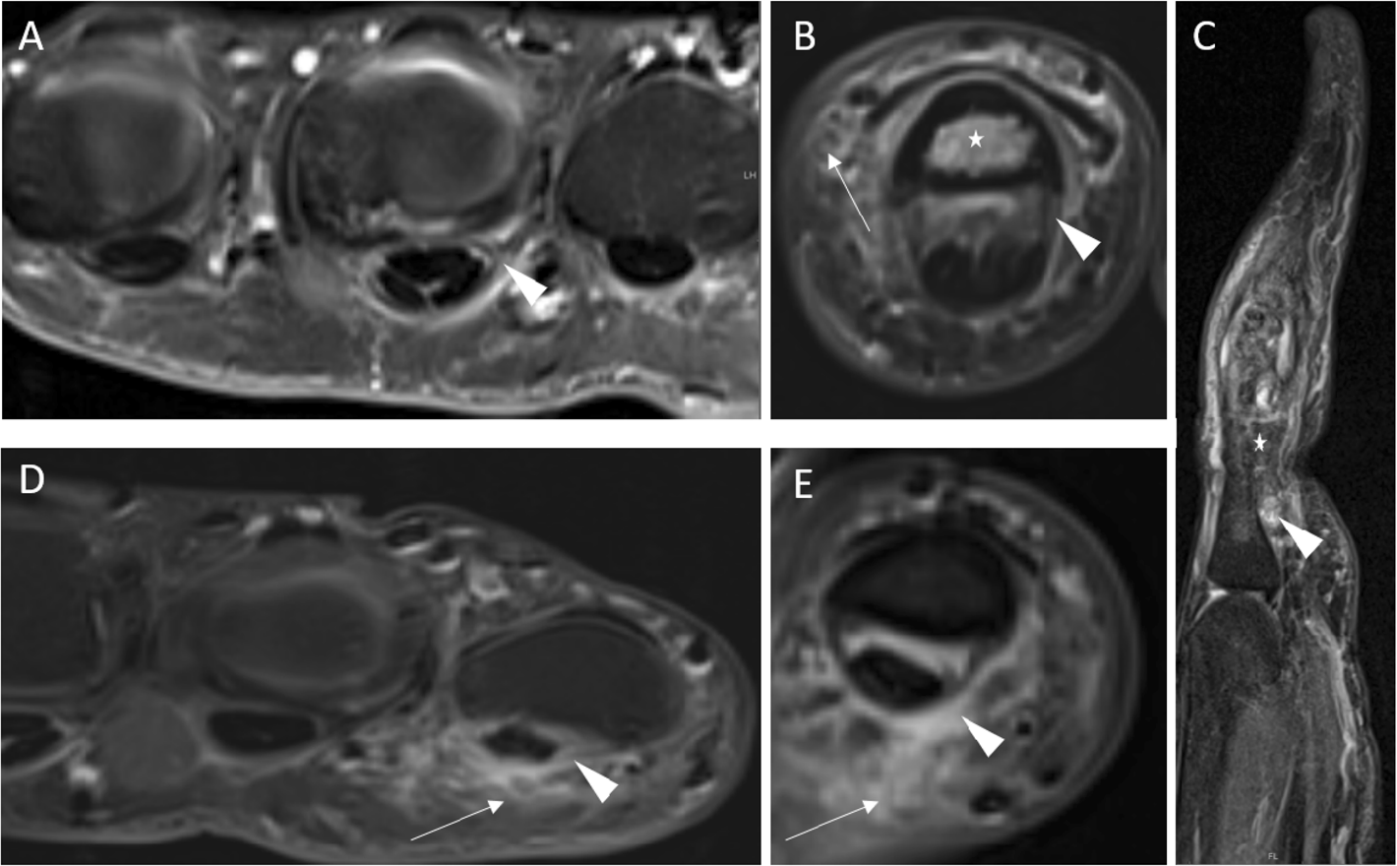
High-resolution MRI of the Hand - Figure 3

MR images were independently read and analyzed by two radiologists (one attending physician [CS], one resident physician [DBA]) with long-term experience in musculoskeletal imaging of > 8 years (CS) and all trained in RAMRIS and PsAMRIS-Scoring according to the OMERACT guidelines [15, 16]. In case of different findings, the readers decided by common consensus with the assisting opinion of a third reader (PS, rheumatologist with 8 years of experience in musculoskeletal imaging). Readers were blinded to the diagnosis of the patients. Flexor tendon pulleys A1 and A2 were analyzed in digits 2 to 5. Each pulley was evaluated regarding its thickness in millimeter and its intrinsic and/or surrounding signal intensity at the radial, ulnar, and volar aspect of each pulley (see Figs. 2 and 3 for typical changes). The PsAMRIS was adapted due to the clear visualization of the pulleys with abnormalities being scored as 0–3 as per PsAMRIS scoring at other sites such as synovium and tenosynovium [21]. Consequently, the score reflected the maximum degree of enhancing and/or hyperintense signals within the pulley complex perpendicular to the pulley at its most inflamed part and scores indicated the absence of any abnormality (score 0), the involvement of < 50% of the pulley thickness (score 1), of ≥ 50–< 100% (score 2), and ≥ its entire thickness (score 3). For each pulley, we took the sum of the radial, ulnar, and volar grading regarding the surrounding and/or intrinsic inflammatory changes and the mean of the radial and ulnar thickness of the pulley itself in millimeter measured at its thickest part. Hence, measurements were not necessarily performed on the same slice. Additionally, PsA and RA patients were evaluated according to PsAMRIS at the MCP, PIP, and DIP (the latter two only in PsA patients) joint level of digits 2–5 for synovitis (score 0–3), flexor tenosynovitis (score 0–3), bone edema (score 0–3), erosion (score 0–10), proliferation (score 0 or 1), and periarticular inflammation (score 0 or 1) [21].

### Statistical analysis

All statistical analyses were performed using SPSS software (IBM, version 22, Armonk, NY, USA). For descriptive analysis, the mean, standard deviation, minimum, and maximum are presented. Mean values were compared with a one-way analysis of variance (ANOVA) and a post hoc Scheffé test. For correlation analyses, Spearman rho correlation coefficient (ρ) was used. Correlation strength was graded as suggested by Cohen [28]: small (< 0.3), moderate (0.3–0.5), and large (> 0.5).

Due to a large number of comparisons between PsA patients, RA patients, and healthy controls, Bonferroni correction was applied, and the level of significance was set to *p* ≤ 0.05/3 = 0.0167. Three patient cohorts were comparatively evaluated and therefore may be considered as separate experiments. In a stricter statistical sense, correction of all 66 sub-experiments (i.e., 3 cohorts, 2 assessed MRI characteristics, and 11 anatomical flexor tendon pulley levels) ought to be performed; however, the increased risk of producing false negatives secondary to overly conservative statistical analyses rendered this option impractical. Accordingly, we decided to use (design-adapted) Bonferroni correction and look for consistent and significant changes of the pulleys as a function of disease entity instead of relying on statistical formalism. Inter- and intra-rater reliability for pulley thickness and pulley inflammatory changes were calculated by two-way mixed intraclass correlation coefficients (single-measure ICC (sICC) for intra-rater and average measure ICC (aICC) for inter-rater reliability).

## Results

### PsAMRIS (sub-scores) at MCP joint level in PsA and RA patients

Descriptive analysis of inflammation sub-scores according to PsAMRIS are illustrated in Table 1 for PsA and in Table 2 for RA patients. Synovitis, flexor tenosynovitis, and periarticular inflammation (extracapsular changes) were commonly found in all PsA and RA patients. Bone edema and bone erosions, on the other hand, were less frequently seen, whereas bone proliferations were rarely detected. Periarticular inflammation (extracapsular changes) and bone erosions were significantly more frequently detected in PsA than in RA patients (*p* = 0.003 and *p* < 0.001) as originally described in hand joints [29]. Regarding all other sub-scores, no significant differences could be detected.

**Table 2:** Mean PsAMRIS values ± standard deviation for each inflammation parameter at MCP joint level in PsA and RA patients

PsAMRIS value	MCP		*p* value
	PsA	RA	
Overall	23.41 ± 4.89	20.45 ± 4.17	0.071
Synovitis	9.18 ± 2.1	10.35 ± 1.87	0.084
Flexor tenosynovitis	4.76 ± 1.44	5.05 ± 1.61	0.572
Periarticular inflammation	6.47 ± 1.66	4.15 ± 2.24	**0.003**
Bone edema	0.6 ± 1.18	0.4 ± 1.27	0.643
Bone erosion	2.29 ± 1.49	0.5 ± 0.76	**< 0.001**
Bone proliferation	0.12 ± 0.33	0	0.163

*p* values for the comparison of means between PsA and RA patients. After (adapted) Bonferroni correction, *p* values < 0.0167 were considered significant and are given in bold type

### Inflammatory changes and thickness of the flexor tendon pulleys

Mean values of inflammatory changes and thickness of the A1 and A2 flexor tendon pulleys in PsA and RA patients and in HC are summarized in Tables 3 and 4. Visualization of flexor pulley changes in Figs. 2 and 3.

**Table 3:** Mean values ± standard deviation and intergroup comparisons of flexor tendon pulley inflammatory changes in PsA, RA, and HC patients

Finger + pulley	PsA	RA	HC	PsA vs RA	PsA vs HC	RA vs HC
	Inflammatory changes			*p* values		
D2 A1	2.82 ± 1.25	2.55 ± 1.43	0.94 ± 1.14	0.82	**0.001**	**< 0.003**
D2 A2	2.06 ± 1.08	1.75 ± 1.55	0.75 ± 1.03	0.77	**0.02**	0.08
D3 A1	3.24 ± 1.59	2.6 ± 1.16	0.69 ± 0.98	0.35	**< 0.001**	**< 0.001**
D3 A2	3.28 ± 1.62	1.85 ± 1.56	0.94 ± 1.03	0.02	**< 0.001**	0.2
D4 A1	2.65 ± 1.37	2.05 ± 1.24	0.5 ± 0.79	0.33	**< 0.001**	**0.002**
D4 A2	2.5 ± 1.12	1.7 ± 1.05	1.13 ± 1.11	0.1	**0.002**	0.32
D5 A1	2.18 ± 1.15	1.5 ± 1.57	0.38 ± 0.70	0.27	**0.001**	**0.036**
D5 A2	2.22 ± 1.27	1.95 ± 1.53	0.69 ± 0.98	0.82	**0.007**	**0.03**
D2–5 A1	10.88 ± 4.30	8.7 ± 3.15	2.5 ± 2.87	0.194	**< 0.001**	**< 0.001**
D2–5 A2	10.06 ± 3.11	7.25 ± 3.65	3.5 ± 3.51	0.031	**< 0.001**	**0.004**
D2–5 A1 + 2	20.65 ± 6.57	15.95 ± 7.01	6 ± 6.3	0.048	**< 0.001**	**< 0.001**

After (adapted) Bonferroni correction, *p* values < 0.0167 were considered significant and are printed in bold type

**Table 4:** Mean values ± standard deviation and intergroup comparisons of flexor tendon pulley thickness in PsA, RA, and HC patients

Finger + pulley	PsA	RA	HC	PsA vs RA	PsA vs HC	RA vs HC
	Pulley thickness in mm			*p* value		
D2 A1	0.86 ± 0.14	0.63 ± 0.10	0.62 ± 0.16	**< 0.001**	**< 0.001**	0.99
D2 A2	0.79 ± 0.13	0.54 ± 0.09	0.6 ± 0.11	**< 0.001**	**< 0.001**	0.45
D3 A1	0.82 ± 0.19	0.6 ± 0.09	0.64 ± 0.11	**< 0.001**	**0.002**	0.76
D3 A2	0.83 ± 0.11	0.58 ± 0.12	0.66 ± 0.18	**< 0.001**	**0.006**	0.269
D4 A1	0.71 ± 0.16	0.6 ± 0.09	0.61 ± 0.09	0.03	0.09	0.94
D4 A2	0.79 ± 0.12	0.58 ± 0.12	0.63 ± 0.13	**< 0.001**	**0.008**	0.54
D5 A1	0.76 ± 0.21	0.58 ± 0.08	0.64 ± 0.13	**0.005**	0.1	0.56
D5 A2	0.71 ± 0.21	0.61 ± 0.08	0.61 ± 0.12	0.13	0.15	1
D2–5 A1	0.79 ± 0.18	0.6 ± 0.09	0.63 ± 0.13	**< 0.001**	**< 0.001**	0.74
D2–5 A2	0.78 ± 0.15	0.58 ± 0.22	0.63 ± 0.14	**< 0.001**	**< 0.001**	0.21
D2–5 A1 + 2	0.79 ± 0.17	0.59 ± 0.19	0.63 ± 0.13	**< 0.001**	**< 0.001**	0.35

After (adapted) Bonferroni correction, *p* values < 0.0167 were considered significant and are printed in bold type

PsA patients had significantly thicker A1 and A2 flexor tendon pulleys in most fingers as compared to RA and HC (overall mean difference compared to RA: 0.19 mm, *p* < 0.001; overall mean difference compared to HC: 0.16 mm, *p* < 0.001).

Additionally, PsA patients showed significantly more inflammatory changes (higher sum-scores) at A1 and A2 flexor tendon pulleys in all fingers as compared to HC (mean difference 14.65, *p* < 0.001). Furthermore, inflammatory changes of all flexor tendon pulleys were higher in PsA than in RA patients (mean difference 4.17; *p* = 0.048). Compared to HC, RA patients had a similar thickness, but more intense inflammatory changes of pulleys (mean difference 9.95, *p* < 0.001). sICC was 0.88 and aICC 0.93.

### Correlation of flexor tendon pulley inflammation and pulley thickness and PsAMRIS (−sub-scores)

Values for Spearman rho correlation coefficients of flexor tendon pulley inflammation and pulley thickness and PsAMRIS (−sub-scores) for PsA and RA patients are displayed in Table 5.

**Table 5:** Spearman rho correlation coefficient (ρ) for the score of inflammatory changes of flexor tendon pulleys (A1 and A2) and PsAMRIS sub-scores, total PsAMRIS, and flexor tendon pulley thickness at D2–5 in PsA and RA patients

	D2		D3		D4		D5	
	PsA	RA	PsA	RA	PsA	RA	PsA	RA
Synovitis	**0.72**	0.49	0.48	0.36	0.4	0.2	**0.83**	0.11
Flexor tenosynovitis	**0.7**	0.49	**0.91**	0.46	**0.7**	0.48	**0.76**	0.41
Periarticular inflammation	0.49	0.49	**0.62**	0.32	**0.77**	0.49	0.6	0.53
Bone erosion	0.37	0.16	0.43	0.31	0.13	na	0.62	0.28
Bone edema	0.52	− 0.34	**0.66**	0.39	0.36	na	0.28	na
Bone proliferation	− 0.03	na	− 0.05	na	− 0.44	na	0.05	na
Overall PsAMRIS	**0.72**	0.46	**0.81**	0.47	0.45	**0.63**	**0.80**	0.46
Pulley thickness	0.23		0.11		0.22		0.07	

*na* = non applicable

After (adapted) Bonferroni correction, *p* values < 0.0167 were considered significant and are printed in bold type

In PsA patients, there was a strong correlation between pulley inflammation and overall PsAMRIS as well as inflammatory PsAMRIS sub-scores (synovitis, periarticular inflammation, and flexor tenosynovitis) at most fingers. A heterogeneous (low to high) correlation was found for pulley inflammation and bone erosion and edema, whereas the correlation of pulley inflammation and bone proliferation was mostly weak. Furthermore, pulley inflammation and thickness showed a weak correlation.

In RA patients, we also found a significant, but weaker, correlation between pulley inflammation and overall PsAMRIS as well as inflammatory PsAMRIS sub-scores as compared to PsA. Correlation coefficients for bone erosion and bone edema were low to intermediate.

## Discussion

Psoriatic arthritis has a prominent entheseal disease component manifesting at the so-called synovio-entheseal complex, that includes flexor tendon pulleys [9]. In this study, we evaluated the value of high-resolution 3 T MRI changes of flexor tendon pulleys in PsA patients for disease detection as well as a distinction from RA and HC using a dedicated 16-channel hand coil.

According to our results, PsA patients had significantly thicker A1 and A2 flexor tendon pulleys than RA patients or HC. Between the latter, on the other hand, there were no significant differences in pulley thickness. These findings of pulley thickening in PsA patients confirm the prior ultrasound studies of Tinazzi et al., who evaluated a small population of patients with PsA, RA, non-arthritic psoriasis (Pso), and healthy individuals [14]. They hypothesized that the distinctive thickening of flexor tendon pulleys in PsA could be due to “deep Koebnerization,” an adaptation of entheses to mechanical stress, according to dermal hyperplasia and thickening in Pso, commonly known as “Koebner phenomena” [14]. Even more recently, Furlan et al. also found thickened A1 pulleys in PsA patients using ultrasound and concluded that pulley thickness could potentially be used to differentiate PsA from other forms of arthritis [30]. In addition, Graceffa et al. also demonstrated entheseal thickening in larger entheses in PsA and Pso patients, and assumed that these distinct changes were triggered by inflammation [31]. Along with our findings, this contributes to the concept of “enthesitis being a hallmark of PsA,” even though we only considered patients with PsA, but not with Pso [25]. Further studies evaluating differences of pulley thickness and inflammation between patients with PsA, skin Pso, and nail Pso would be of great interest. Our high-resolution MRI study is the first to demonstrate these distinct PsA features using an alternative imaging method to sonography. Both modalities, ultrasound and MRI, are validated high-resolution imaging modalities for the evaluation of inflammatory joint changes in PsA such as thickening and inflammatory edema of tendons and ligaments. As compared to ultrasound, MRI is highly reproducible and can detect additional disease-related imaging features that ultrasound would miss such as bone marrow edema [32, 33]. MRI, however, is more time-consuming, less dynamic, more expensive, and reliant on considerably larger infrastructural requirements [33]. In addition, this study demonstrated that flexor tendon pulleys of PsA patients were not only thicker than those of HC and RA patients, but also showed more frequent and intense inflammatory changes in their course and at their insertion sites. However, these findings were only numerically (not statistically) significant regarding RA patients. In 2015, Tan et al. had already demonstrated that MRI inflammatory changes of finger entheses were more frequent and severe in PsA patients than in HC, but they only evaluated fingers with acute dactylitis and did not use a dedicated 16-channel hand coil as we did in this study [13]. In particular when assessing small soft tissue structures as the A1 and A2 pulleys whose mean thickness we determined as 0.63 mm in healthy individuals (Table 4), MRI technique needs to be optimized regarding sequence parameter settings and coil and scanner configurations enable true high-resolution imaging. Consequently, we achieved in-plane pixel dimensions of 0.3 × 0.3 mm^2^.

Our findings show inflammation of pulleys at both dactylitic and non-dactylitic fingers and, therefore, expand the value of pulley involvement beyond the scope of dactylitis. Potentially this is partly due to a higher image resolution using a 16-channel hand coil in the present study and suggests that the non-dactylitic tenosynovitis in PsA is also linked to pulley inflammation.

Furthermore, despite similar findings regarding pulley thickness, our data also showed more pulley-associated inflammation in RA patients than in HC. Considering that RA is primarily a synovial disease that is known to secondarily affect adjacent structures, such as flexor tendons, the involvement of entheses, and hence pulleys, seems evident. However, previous studies have shown that RA, despite involving the synovio-entheseal complex, primarily affects its synovial aspect; PsA on the other hand shows a stronger affection of its entheseal component [34]. Along with our findings of more intense and more frequent involvement of pulleys in PsA than in RA, this supports the idea of PsA being more predominantly an entheseal and extracapsular disease and contributes to the distinction of the two entities. In addition, it remains for future studies to assess whether these differences between both entities become more distinct in more distal pulleys, i.e., A 3–5. Since early diagnosis and treatment is pivotal for a better outcome in both entities and treatment options are increasingly diverging due to the development of biological and targeted synthesized disease-modifying anti-rheumatic drugs (bDMARD and tsDMARD), an early distinction between the two would be highly beneficial.

The presented data shows only a weak correlation between inflammatory changes and thickness of A1 and A2 flexor tendon pulleys in PsA patients. This could be due to the patient population of non-early PsA patients that show side-by-side signs of acute inflammation and post-inflammatory changes in different joints/entheses. Thus, on the one hand, thickened pulleys could be associated with acute inflammation, while, on the other hand, being a result of previous inflammation. Baraliakos et al. also demonstrated that chronic changes of entheses can occur without inflammation present in patients with peripheral spondyloarthritis [35]. Despite a weak correlation between pulley thickness and inflammation, we found a strong correlation between pulley inflammation and overall PsAMRIS, flexor tenosynovitis, periarticular inflammation and synovitis, and to a lesser extent with bone erosion and bone edema sub-score. It is worth noting though that our population of PsA patients’ scores for the PsAMRIS item “periarticular inflammation” were higher than in previous studies [24, 36]. However, in contrast to these studies, we have recruited patients who fulfilled the CASPAR criteria and additionally suffered from dactylitis of at least one digit. Since periarticular inflammation is considered an imaging core feature of dactylitis, our study design might have led to an overrepresentation of that particular PsAMRIS item. PsAMRIS and its sub-scores are validated tools for the detection and monitoring of disease-related joint changes. Therefore, inflammatory changes of pulleys could also be appropriate for the evaluation of disease-driven joint involvement [24]. In early 2019, Mathew et al. introduced a preliminary enthesitis-based MRI scoring system, named “Heel Enthesitis Scoring System” (HEMRIS) that emphasizes the value of entheseal changes for the diagnosis and monitoring of PsA [26].

The following limitations to this study must be considered when interpreting its results; as our study has only a small patient cohort size, our results have to be considered preliminary. Accordingly, further investigations with larger patient cohorts are required. The mean disease duration of the PsA and RA study population differed by approximately 109 weeks (RA 11, PsA 120 weeks). Applying current definitions, we hence compare non-early PsA with early RA populations (early RA: disease duration < 12 months; early PsA: disease duration < 24 months) [37, 38]. Thus, the comparability of both populations is potentially limited and the differences regarding inflammatory changes of the pulleys might be affected by differences in disease duration; however, because PsA is a very heterogeneous disease, the definition of disease onset and, therefore, an “early” patient population is challenging. In addition, enthesitis is a sign of acute inflammation, and hence not limited to advanced disease stages. Since the evaluation of enthesitis, namely inflammatory changes of flexor tendon pulleys, was our main goal, we consider the divergent disease duration only a minor limitation. However, in future investigations, study populations with a similar disease duration are required.

Even though the readers were blinded to the patients’ diagnosis, different field-of-views of certain sequences might have corrupted proper blinding.

Unfortunately, we did not systematically count swollen and tender joints in a per-digit and per-joint manner at the time of recruitment for the MRI studies. Thus, we cannot further elucidate the association of inflammatory pulley changes and their clinical manifestation. Therefore, future studies are required that further investigate the association of imaging features and clinical findings. MRI generally has a limited spatial resolution. Since flexor tendon pulleys frequently have a thickness of < 1 mm there is an increased risk of measuring inaccuracy due to partial volume effects, which may bring about substantial inter- and intra-rater variability. However, our findings show a good intra- and inter-rater reliability that is in coherence with previous studies regarding MRI measurements of pulleys and may also be the result of the optimized measurement setup that allows high-resolution imaging [39].

## Conclusion

In conclusion, the assessment of high-resolution MRI changes of flexor tendon pulleys using a 16-channel hand coil could be used for disease detection in PsA and is potentially beneficial for the distinction from RA and HC.

## Data Availability

The datasets used and/or analyzed during the current study are available from the corresponding author on reasonable request.

## Abbreviations

ACR: American College of Rheumatology
ADAM: Analysis of the DActylic Melange
aICC: Average measure ICC
bDMARD: Biological DMARD
CAR-ERA: Cartilage in early RA
CASPAR: Classification criteria for psoriatic arthritis
DIP: Distal interphalangeal
DMARD: Disease-modifying drug
EULAR: European League Against Rheumatism
HC: Healthy control
ICC: Intraclass correlation coefficient
MCP: Metacarpophalangeal
MRI: Magnetic resonance imaging
MTX: Methotrexate
OMERACT: Outcome measures in rheumatoid arthritis clinical trials
PD: Proton density
PIP: Proximal interphalangeal
PsA: Psoriatic arthritis
PsAMRIS: Psoriatic arthritis magnetic resonance imaging score
RA: Rheumatoid arthritis
RAMRIS: Rheumatoid arthritis magnetic resonance imaging score
SE: Spin echo
sICC: Single measure ICC
STIR: Short tau inverse recovery
T: Tesla
T2T: Treat to target
TE: Echo time
TR: Relaxation time
tsDMARD: Targeted synthetic DMARD
TSE: Turbo spin echo
